# SMA-10 Is a Non-Canonical Member of the TGF-β Sma/Mab Pathway and Immunity Regulator via the DAF-2 Insulin Receptor in *Caenorhabditis elegans*

**DOI:** 10.3390/ijms22020638

**Published:** 2021-01-11

**Authors:** María Pilar de Lucas, Marta Jiménez, Paloma Sánchez-Pavón, Alberto G. Sáez, Encarnación Lozano

**Affiliations:** Unidad Funcional de Investigación de Enfermedades Crónicas, Instituto de Salud Carlos III, Majadahonda, 28220 Madrid, Spain; mpdelucas@isciii.es (M.P.d.L.); marta.jimenezsanchez90@gmail.com (M.J.); paloma.sanchezpavon@gmail.com (P.S.-P.); agsaez@gmail.com (A.G.S.)

**Keywords:** *sma-10*, TGF-β Sma/Mab, TGF-β signalling, IIS pathway, *daf-2*, innate immunity

## Abstract

Transforming growth factor β (TGF-β) signalling pathways are highly conserved across metazoa and play essential roles not only during development but also in adult tissue maintenance. Alterations of these pathways usually result in a plethora of pathologies. In the nematode *Caenorhabditis elegans*, the TGF-β Sma/Mab (small/male abnormal) pathway regulates various worm phenotypes such as body size, immune response, ageing, matricide and reproductive span. SMA-10 has been described as a positive modulator of worm body size through the TGF-β Sma/Mab pathway. To better understand if SMA-10 is a core component of the pathway, we use gene epistatic analysis to assess the contribution of SMA-10 to various phenotypes regulated by TGF-β Sma/Mab. We confirm that SMA-10 controls body size and find that it also affects the matricide and reproductive span of the nematodes. However, neither male tail formation (previously reported) nor ageing appeared altered. Lastly, although null *sma-10* worms are more susceptible to *Pseudomonas aeruginosa* infections than wild-types, this response does not depend on TGF-β Sma/Mab but on the insulin receptor DAF-2. We also show that the expression of *sma-10* in either hypodermis or intestine fully rescues the wild-type immune response. Our results contribute to understanding the role of SMA-10 as a context-dependent component of TGF-β Sma/Mab, and reveal a function of SMA-10 in immunity in association to the Insulin/insulin-like growth factor signalling (IIS) pathway.

## 1. Introduction

The transforming growth factor β (TGF-β) superfamily controls numerous cellular processes during development and tissue homeostasis in adults. In humans it comprises more than 30 different secreted molecules, with common properties in their molecular mechanisms of action, also shared across metazoa: they function by binding to transmembrane serine/threonine kinase receptors. Upon ligand recognition, a heterotetrameric receptor complex is formed between two type I and two type II receptors. Type II protein, constitutive active, phosphorylates type I, and allows the activation of downstream Smad transcription factors which regulate gene expression. Despite the evolutionary conservation of this “canonical” signalling pathway across tissues, cellular types, and throughout the animal kingdom, there is a wide variety of cellular outcomes associated to TGF-β in humans. This multifunctional nature of TGF-β has been referred to as its “enduring mystery” [1]. It is understood that different cellular contexts will not only determine the exact combination of available ligands-receptors, but also the presence of particular accessory receptors or co-receptors that will facilitate or inhibit ligand recognition and the subsequent biochemical signalling. Thus, for instance, the accessory receptor β-glycan can either present or inhibit TGF-β to its receptors, depending on the particular molecular context, implying different cellular responses (reviewed by [1]). Therefore, it is important to determine how the associated proteins to the TGF-β signalling system contribute to the various phenotypic outcomes.

As expected, compared to vertebrates, the nematode *Caenorhabditis elegans* shows a simplified version of the TGF-β signalling. This may facilitate an easier understanding of the multifunctional bases of TGF-β signalling. In *C. elegans*, one of the best known TGF-β pathways controls body size and male tail development. It is known as the TGF-β Sma (small)/Mab (male abnormal) pathway, or simply Sma/Mab, but it also controls other phenotypes later identified such as the immune response, longevity, reproductive span and matricide [2,3]. The ligand of TGF-β Sma/Mab, DBL-1, binds to the SMA-6 (type I) and DAF-4 (type II) receptors and the signal is transmitted through the Smad transcription factors SMA-2, SMA-3 and SMA-4. As in mammals, several proteins that modulate the strength of the DBL-1 signal have been reported: DRAG-1 [4], SMA-10 [5], UNC-40 [6], and others [3]. *sma-10* mutants were initially isolated in a forward genetic screen for small size mutants [7]. From this screen, Gumienny and colleagues identified and characterized *sma-10* as a Sma/Mab component indispensable for body size acquisition [5]. *sma-10* is conserved in *Drosophila* (*lambik*) and vertebrates (LRIG1, LRIG2 and LRIG3; Leucine-rich repeats and immunoglobulin-like domains proteins). All these genes encode proteins with shared structural features: 16-17 tandem leucine-rich repeats, three immunoglobulin-like domains, a transmembrane domain and a cytoplasmic tail variable in length [8,9]. The expression of *Drosophila lambik* can rescue the wild-type body size in *sma-10* mutants, pointing out to an evolutionary conservation of function, which has also been related to the endocytic trafficking of the receptor as a way to transmit the TGF-β signalling into the cytoplasm [10].

In this work, we examined how widespread is the involvement of SMA-10 in TGF-β Sma/Mab, to understand to what extent SMA-10 is a context-dependent component of this pathway. We found that SMA-10 is independent from Sma/Mab in half of the explored phenotypes. Additionally, we found that for one of those phenotypes, innate immunity, SMA-10 depends, on DAF-2 rather than on Sma/Mab, which is the only known receptor of the Insulin/insulin-like growth factor signalling (IIS) [11].

## 2. Results

### 2.1. C. elegans SMA-10 Regulates Growth through Transforming Growth Factor β (TGF-β) Small/Male Abnormal (Sma/Mab) via Endoreduplication

**Figure 1:**
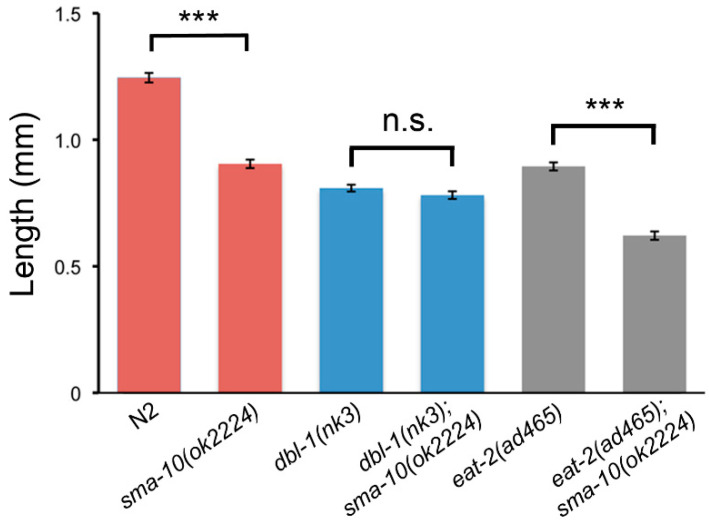
Adult body length of N2, *sma-10(ok2224)*, *dbl-1(nk3)*, *dbl-1(nk3);sma-10(ok2224)*, *eat-2(ad465)* and *eat-2(ad465);sma-10(ok2224)* worms. At least 20 animals from a minimum of two independent experiments were synchronised and measured as described in Methods, and its final length were plot. Error bars indicate 95% confidence intervals. Not significant (n.s.) and significant statistical differences (*** *p* < 0.001) are indicated.

The discovery of Kekkon5 as a new modulator of BMP (bone morphogenetic protein; of the TGF-β superfamily) signalling in *Drosophila* [12] prompted us to search for the *C. elegans* orthologs. We found that Kekkon5 shared 24% identity to, at that time, the uncharacterised protein T21D12.9; later identified as SMA-10 [5]. We used RB1739, a mutant strain with genotype *sma-10(ok2224)* and available at Caenorhabditis Genetics Center, to analyse its phenotype. This mutant contains a 906 nucleotide deletion that completely removes part of the exon 9 and the whole exon 10, which results in SMA-10 lacking the three Ig-like motifs, the transmembrane domain and the cytoplasmic tail (Figure S1); suggesting that the *sma-10(ok2224)* allele is null. We observed that animals with this deletion are small and, through epistatic analysis, verified that *sma-10* controls body size through the TGF-β Sma/Mab pathway, as later shown for defective worms carrying other *sma-10* deletion alleles [5]. More specifically, our results showed that double mutants lacking both SMA-10 and the ligand DBL-1 display a body size similar to DBL-1 defective worms (Figure 1). In contrast, double mutants for SMA-10 and EAT-2, a channel subunit that controls pharyngeal pumping and thus results in small and starved worms [13], independently of Sma/Mab, produce significantly smaller animals than either single mutant (*dbl-1(nk3)*, *eat-2(ad465)*). In short, and in agreement with previous results [5], *sma-10* specifically interacts with the Sma/Mab pathway in relation to body size regulation.

Much of the effect of Sma/Mab on body size in *C. elegans* is exercised via the endoreduplication of the hypodermal nuclei [14]. Therefore, we checked the ploidy content of *sma-10(ok2224)* worms. These showed lower hypodermal ploidy levels than N2 and slightly higher hypodermal ploidy levels than *dbl-1(nk3)* (Table 1). Double mutants *dbl-1(nk3)*;*sma-10(ok2224)* behaved indistinguishably from *dbl-1(nk3)* or *sma-10(ok2224)*. These results support the notion that the body size displayed by *sma-10(-)* mutants is controlled by their hypodermal ploidy, as previously described for other TGF-β Sma/Mab pathway mutants [14].

### 2.2. SMA-10 Is Dispensable for Male Tail Formation

**Figure 2:**
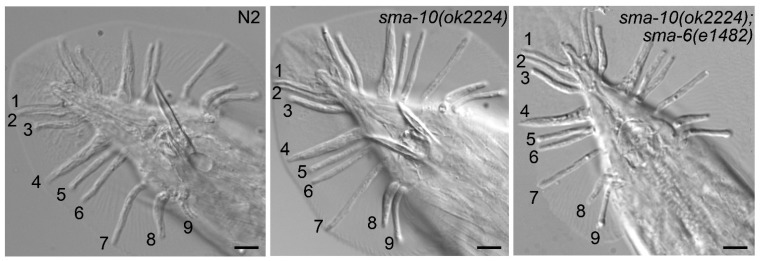
Male tail development is not modulated by SMA-10. Young adult males were anesthetized with 1% sodium azide and mounted in 2% agar pads. Worms were observed at ×1000 amplification under Nomarski interference contrast optics with an Axio Zeiss Imager.A1 microscope. Scale bar represents 10 μm. Numbers denote the position of the rays.

To understand how prevalent is the participation of SMA-10 in various functions assigned to TGF-β Sma/Mab, we then examined the possible involvement of *sma-10* in relation to male tail formation, a known function of *C. elegans* Sma/Mab. Our results, using *sma-10(ok2224)*, coincide with a previous report [5] in which different loss-of-function alleles of *sma-10* did not affect male tail development: no obvious morphological defects were observed in rays or spicules (Figure 2). We also observed that *sma-10(ok2224)* mutant males were able to copulate normally, as opposed to other Sma/Mab mutant males.

One possibility is that tail development was less sensitive to the TGF-β (DBL-1) dose, implying that only a significant decrease in the signal would perturb normal development. To check this, we chose the hypomorphic mutation for the receptor *sma-6(e1482)*, which is not defective for male tail formation (null *sma-6* mutants are) [15], and generated double mutants that carried *sma-10(ok2224)* and *sma-6(e1482)*. If *ok2224* were able to decrease the Sma/Mab signalling in relation to male tail formation, it is likely that the *sma-10(ok2224);sma-6(e1482)* worms would have male tail defects, but again, we could not observe any of those defects (Figure 2). In our view, this suggests that SMA-10 does not play indeed any obvious role in the development of male tails.

### 2.3. Longevity Is Not Affected by SMA-10 Expression

**Figure 3:**
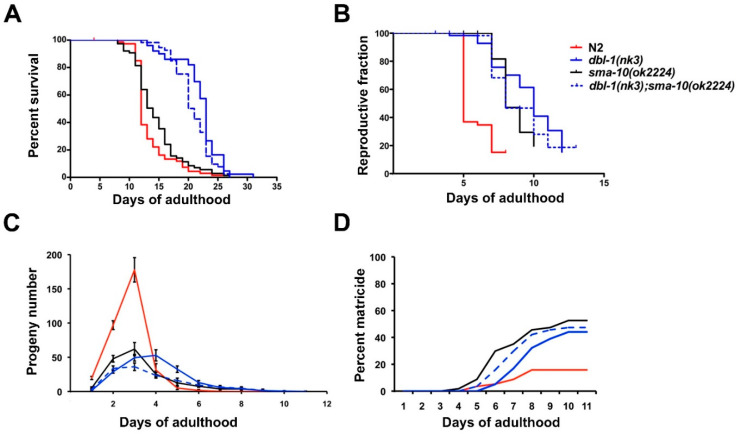
Role of SMA-10 in longevity, fecundity and matricide. Data shown are from one representative experiment. (**A**) Kaplan–Meier plot shows the fraction of animals that survived overtime. Strains were grown at 20 °C in Nematode Growth Medium(NGM) plates with OP50 and 50 mM FUdR. *sma-10(ok2224)* worms, in contrast to *dbl-1(nk3)* and *dbl-1(nk3);sma-10(ok2224)*, do not show a significant difference with respect to N2. (**B**) Kaplan–Meier plot with the fraction of animals reproductively active overtime. Reproduction span is similar in *dbl-1(nk3)*, *sma-10(ok2224)* and *dbl-1(nk3);sma-10(ok2224)* and significantly longer than in N2. (**C**) Deposition of eggs peaks around day three of adulthood in N2, rapidly decreasing thereafter; in contrast, self-fertilized mutants of *sma-10* and *dbl-1* not only have a longer deposition span but show a lower overall fertility. (**D**) Cumulative percentage of animals with matricide (caused by internal progeny hatching). Matricide rate increases similarly with age in defective mutants of *dbl-1*, *sma-10* and their double mutant, unlike the slower rate of the wild-type N2.

In relation to longevity, another trait affected by TGF-β Sma/Mab (null mutants live longer than wild-type worms [16]), *sma-10(ok2224)* worms do not show a significant difference compared to N2 (median survival 14 vs. 12 days, respectively, *n* = 75 both, *p* = 0.07; Figure 3A). As expected, *dbl-1(nk3)* survived longer than N2 (23 days, *n*= 50, *p* < 0.0001). We also noticed that *dbl-1(nk3);sma-10(ok2224)* were similar to *dbl-1(nk3)* (21 days, *n* = 55, *p* = 0.017; Figure 3A). These results show that worm survival span is not significantly affected by SMA-10 expression.

### 2.4. Reproductive Ageing Is Regulated by SMA-10

Another interesting role of TGF-β Sma/Mab is its strong influence on reproductive ageing. Multiple aspects of the reproductive process underlie this trait in animals (embryo integrity, oocyte fertility, chromosome segregation fidelity, and others) [16,17], but they can be globally assessed by recording the temporal pattern of viable egg deposition. Thus, we observed that the reproductive span of *sma-10(ok2224)* hermaphrodites is similar to both, *dbl-1(nk3)* and *dbl-1(nk3);sma-10(ok2224)* (8, 10 and 8 days, respectively, *n* ≈ 60 each, *p* > 0.1), and longer than N2 (5 days, *n* = 58, *p* < 0.0001; Figure 3B). We also noticed that *sma-10(ok2224)* showed a much less pronounced peak of egg deposition, because it produces a smaller number of total eggs in an extended time (169 vs. 332 eggs of N2), and similarly to *dbl-1(nk3)* (189) and *dbl-1(nk3);sma-10(ok2224)* (135; *n* = 30 for each strain; Figure 3C). We conclude that SMA-10 is required for the Sma/Mab pathway to control reproductive ageing.

### 2.5. Matricide Rate Is Similar in sma-10 Defective Worms Than in dbl-1 Mutants

TGF-β Sma/Mab mutants exhibit a highly penetrant late egg-laying defect with internal hatching, i.e., matricide or “bagging”, at the end of their reproductive period [16]. Therefore, we addressed whether *sma-10* mutants also exhibit this property: *sma-10(ok2224)* presents around 50% matricide, similarly to *dbl-1(nk3)* (44%) and *dbl-1(nk3);sma-10(ok2224)* (47%) (Figure 3D). Therefore, SMA-10 participates in the TGF-β Sma/Mab signalling leading to the matricide phenotype.

### 2.6. SMA-10 Plays a Role in Immunity Independently of TGF-β Sma/Mab Pathway

**Figure 4:**
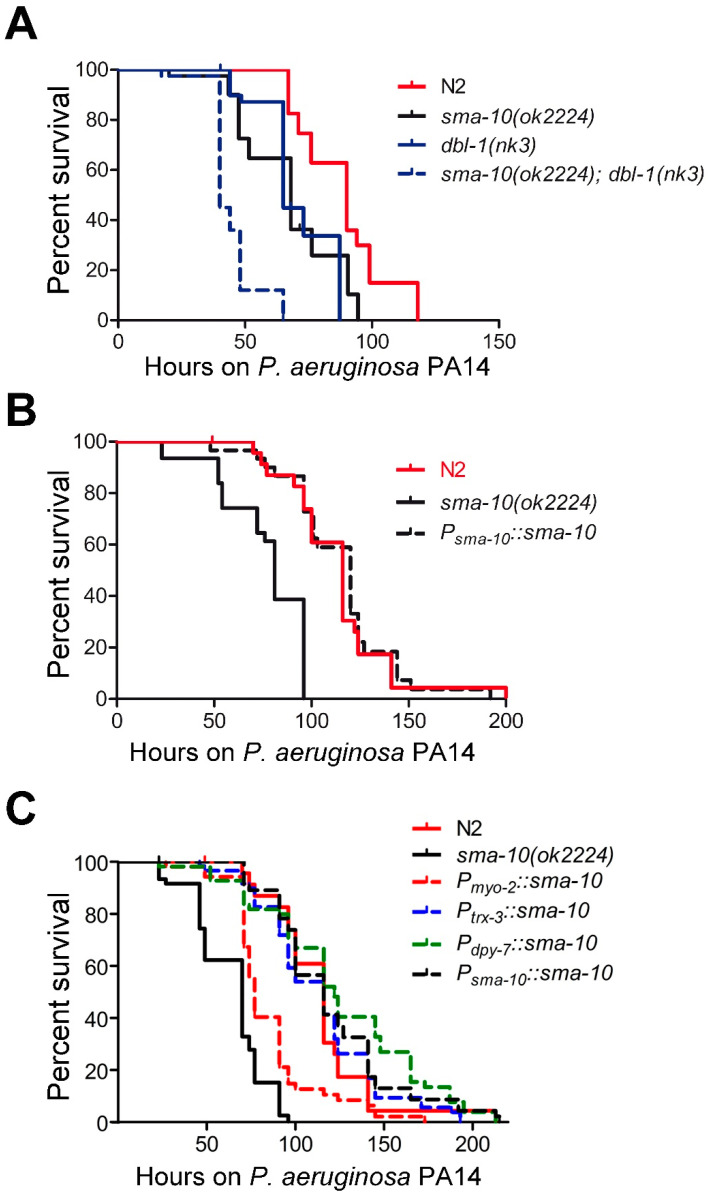
The expression of SMA-10 in intestine and hypodermis is required for the immune response to infection by *Pseudomonas aeruginosa* (strain PA14) independently from DBL-1. Kaplan–Meier plot was used to show the fraction of animals that survived overtime to PA14 infection; data shown are from one representative experiment. (**A**) *sma-10(ok2224)* mutants are more sensitive to PA14 infection than the wild-type, whereas *dbl-1(nk3);sma-10(ok2224)* animals survive even less than *sma-10(ok2224)* or *dbl-1(nk3)*, suggesting an independent function of SMA-10 with respect to DBL-1 in relation to immunity. (**B**) The entopic overexpression of SMA-10 (with its own promoter) rescued the deficient response to PA14 infection of *sma-10(ok2224)* worms, indicating its unequivocal role in immunity. (**C**) Wild-type SMA-10 expressed from endogenous, intestinal (*P_trx-3_*) or hypodermal (*P_dpy-7_*) promoters fully rescued the *sma-10(ok2224)* mutant phenotype, whereas a restricted pharyngeal (*P_myo-2_*) expression only produces a partial rescue.

The involvement of the Sma/Mab pathway in pathogen resistance [18] prompted us to examine the influence of SMA-10 on bacterial infection resistance. We focused on the pathogenic effect of *Pseudomonas aeruginosa* (strain PA14), arguably the most common pathogen in immunity studies of worms. We observed that *sma-10(ok2224)* mutants displayed enhanced sensitivity to killing by PA14 with respect to N2 (Figure 4A; Table S2). We confirmed that the enhanced susceptibility to PA14 infection was due to *sma-10* deficiency, as the expression of a 7 kb genomic DNA encompassing from the promoter region to the 3’UTR of *sma-10* was able to completely rescue the lower survival rate of *sma-10(ok2224)* (Figure 4B; Table S2).

On the other hand, animals deficient in both *dbl-1* and *sma-10* further enhanced the susceptibility to PA14 (Figure 4A; Table S2), suggesting that SMA-10 plays an immunological role independently of the Sma/Mab pathway.

#### 2.6.1. SMA-10 Functions in Hypodermis and Intestine to Regulate Immunity

SMA-10 is expressed mainly in the pharynx and intestine, and weakly in hypodermis [5]. In order to identify the tissues where SMA-10 exerts its protective function against PA14 infection, we microinjected the genomic *sma-10* fragment driven by *myo-2* [19], *dpy-7* [20] or *trx-3* [21] promoters in *sma-10(ok2224)* mutants to express SMA-10 in pharyngeal muscles, hypodermis or intestine, respectively. Our results showed that the expression of SMA-10 in intestine or hypodermis, but not in the pharynx, fully recovered the native immune response of *sma-10(ok2224)* (Figure 4C; Table S2). This suggests that intestine and hypodermis contribute, in an independent manner, to the antimicrobial response mediated by SMA-10.

#### 2.6.2. Pathogenic Defence via SMA-10 Runs in Parallel to Various Immunity Pathways

In an effort to identify the molecular mechanisms underlying the observed role of SMA-10 in PA14-mounted defence, we generated double mutant worms carrying deleterious mutations in both *sma-10* and each of the following genes: *sek-1* (Mitogen-Activated Protein Kinase Kinase, of the p38 MAPK pathway [22]); *bar-1* (β-catenin, component of the innate immune response [23]); *fshr-1* (leucine-rich repeat receptor [24]); and *tol-1* (toll-like receptor [25]).

**Figure 5:**
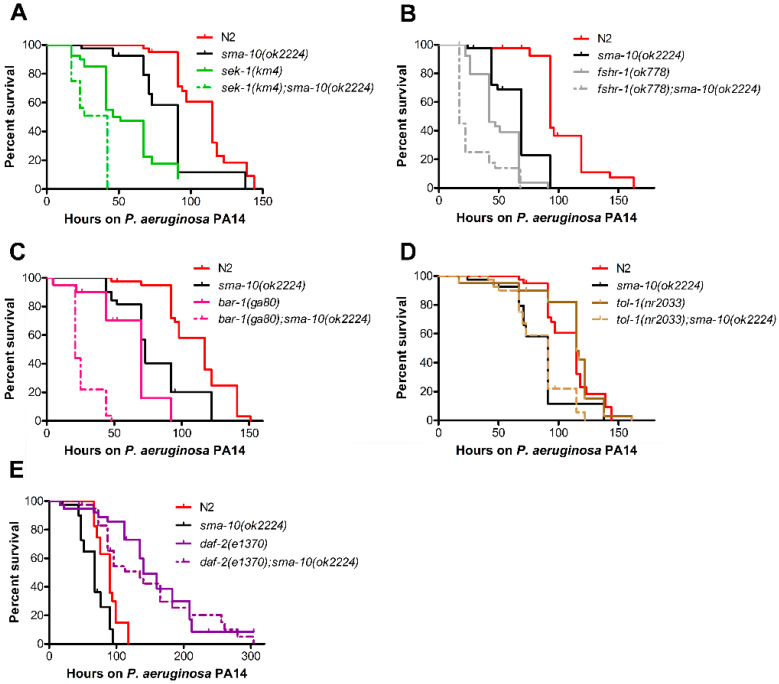
The immunological role of SMA-10 is independent of other proteins and pathways with antimicrobial properties, with the exception of DAF-2. Sensitivity of *sma-10(ok2224)* mutants to *Pseudomonas aeruginosa* (strain PA14) infection was compared to the correspondent double mutants with the following loss-of-function alleles: (**A**) *sek-1(km4)*, (**B**) *fshr-1(ok778)*, (**C**) *bar-1(ga80),* (**D**) *tol-1(nr2033)*, and (**E**) *daf-2(e1370)*. Kaplan–Meier plot was used to show the fraction of animals that survived overtime to PA14; data shown are from one representative experiment. Double mutants are even more sensitive than either single mutant, with the exception of *daf-2(e1370);sma-10(ok2224)* (panel E). Nematodes *daf-2(e1370);sma-10(ok2224)* are as resistant to PA14 infection as *daf-2(e1370)*, suggesting that SMA-10 acts upstream of DAF-2 within the same signalling pathway.

We first confirmed that the *sek-1(km4)*, *fshr-1(ok778)* and *bar-1(ga80)* mutants were more sensitive to killing by PA14 than N2. Then, each of them in association with *sma-10(ok2224)*, became even more vulnerable (Figure 5A–C, Table S3), suggesting that the immunity signal of SMA-10 acts in parallel to the p38 MAPK, *fshr-1* and *bar-1*/Wnt pathways.

In the case of TOL-1, the only toll-like receptor identified in *C. elegans*, its function in immunity is controversial. Pujol and colleagues believed that *C. elegans* TOL-1 does not play any direct role in the defence response [26]. However, another report states the antipathogenic involvement of TOL-1 [25]. We found that *tol-1(nr2033)* mutants behave generally as wild-type animals in terms of PA14 susceptibility (Figure 5D, Table S3). The addition of the *sma-10* mutation in a *tol-1(nr2300)* background induces a *sma-10*-like response to PA14 infection (Figure 5D, Table S3). Therefore, SMA-10 also operates independently of TOL-1 pathway in the immunological response to PA14.

#### 2.6.3. SMA-10 Modulates Immunity via DAF-2 Insulin Receptor

Another important immunity route is the insulin/insulin-like growth factor signalling (IIS) pathway. Having discarded all the other signalling circuits, we asked whether the role of SMA-10 in immunity might be associated to IIS. Many proteins are part of this complex pathway, but DAF-2 is the only protein constituting its cell membrane receptor. Loss-of-function mutants of *daf-2*, such as *daf-2(e1370)* (in contrast to *sma-10(ok2224)*) are more resistant to *Pseudomonas aeruginosa* and other pathogenic bacteria than wild-type worms [27]. Surprisingly, *sma-10(ok2224);daf-2(e1370)* animals behaved as *daf-2(e1370)* (Figure 5E, Table S3). This indicates that *sma-10* role in immunity is dependent on the *daf-2*/insulin molecular pathway and that *daf-2* is, with respect to that phenotype, epistatic to *sma-10*.

## 3. Discussion

We studied the extent to which the transmembrane protein SMA-10, part of the TGF-β Sma/Mab network in *C. elegans*, is responsible for the various phenotypes regulated by that pathway. We found that *sma-10* is required as part of the Sma/Mab signalling in body size [5], reproductive ageing and matricide, and that its absence has no significant effect on male tail formation [5] and longevity; and that the contribution of *sma-10* to the innate immunity does not rest on Sma/Mab. Therefore, it appears that SMA-10 is only required by half of the examined functions of Sma/Mab in *C. elegans*. This suggests that SMA-10 does not belong to the canonical set of proteins most commonly required for every role in which Sma/Mab is active, but to the non-canonical set the participation of which is reserved to only some of those functions. Our results are consistent with the notion acquired from mammals by which TGF-β pathways are especially shaped by the cellular contexts in which they act, giving rise to a variety of phenotypic effects, sometimes even opposite to each other [1].

One way to interpret our results would be that, as has been proposed for male tail formation, some Sma/Mab functions may require lower levels of signalling than for example body size [15]. Hence, *sma-10(ok2224)* might be able to decrease the Sma/Mab signal effectively enough to be noticed in body size reduction but not in male tail formation. However, our results using the hypomorphic mutation *sma-6(e1482)* in conjunction with *sma-10(ok2224)*, which give rise in the double mutant to wild-type male tails, just like with either mutation alone, argue against this “threshold hypothesis” (Figure 2).

We rather favour the idea that SMA-10 is a protein which interacts with TGF-β Sma/Mab in a restricted spatiotemporal manner due to differences in gene expression. We examined the predicted expression of Sma/Mab genes, including *sma-10*, across 76 cell types (https://worm.princeton.edu/; Kaletsky et al., 2018), but did not observe any obvious pattern of gene expression clearly coincident with our results. However, we noticed some intriguing differences in the expression of *sma-6*/*daf-4* (receptor) versus *sma-10* across several types of head neurons and gonadal sub-tissues that perhaps could explain some of the functional differences between the *sma-10* and the *sma-6*/*daf-4* mutants.

It is perhaps also possible that the variety of actions of SMA-10 depend on the differential regulation of other Sma/Mab associated proteins. The list of recognized modulators at the ligand-receptor level has grown in recent years offering us an increasingly complex, although preliminary, map of interactions [3,28,29]. It might be that differences in the expression of those modulators also interacting with SMA-10 were responsible for the variety of Sma/Mab phenotypes attributed to SMA-10. Thus it is of interest not only to study the various phenotypes corresponding to those modulators, but to use a variety of Sma/Mab phenotypes to look for new regulators. We already know that some of those associated proteins do not determine male tail formation (i.e., SMA-10 [5], and this study; DRAG-1 [4]; SMOC-1 [30]), but to our knowledge, no other phenotypes (apart from male tail, body size and mesoderm formation) are investigated in relation to those associated proteins.

Although longevity and reproductive ageing are genetically coupled, a certain degree of dissociation between both has been described; so whereas mutations at the Sma/Mab pathway tend to affect both ageing processes, they alter reproductive span to a larger extent than life span [16]. That distinction is evident in our results with *sma-10(ok2224)*, with a reproductive ageing similar to *dbl-1(nk3)* but, contrary to *dbl-1(nk3)*, with a missing effect on longevity (Figure 3). Similar results were observed with two other Sma/Mab genes, *sma-3* and *sma-9* [16]. We noticed that the matricide was somewhat higher in *sma-10(ok2224)* than in *dbl-1(nk3)*, and both notably larger than in N2 (Figure 3D). As indicated by [16], this may be due to lack of concomitance between somatic and reproductive ageing, forcing ageing mothers to give birth later in life, and therefore impinging a higher reproductive cost (matricide) in *sma-10(ok2224)* compared to *dbl-1(nk3)*, and especially in comparison with the wild-type strain N2.

As shown, TGF-β Sma/Mab plays a protective immunological role independently from SMA-10 (Figure 4A). This result reinforces the hypothesis that the Sma/Mab involved in immunity may be non-canonical; since the DBL-1-dependent upregulation of the antimicrobial peptide caenacin-2 occurs similarly in *sma-2(-)* or *sma-4(-)* as in N2 [31]. Both a non-canonical DBL-1-dependent immunological pathway and a SMA-10’s antipathogenic role independent from Sma/Mab are consistent with the idea that innate immunity and organismal growth are independent physiological processes in worms.

We also found that the lack of the insulin receptor DAF-2 eliminates the sensibility of SMA-10 defective worms to *P. aeruginosa* PA14 (Figure 5E). This places DAF-2 genetically downstream of SMA-10 and suggests that the immunological role played by SMA-10 is related to the IIS pathway. That agrees well with the fact that both proteins, SMA-10 and DAF-2, are expressed in the intestine and hypodermis [5,32,33,34], and that SMA-10’s expression in either intestine or hypodermis is enough to restore its protective role against PA14 in a *sma-10(ok2224)* background (Figure 4C). Given that SMA-10 is a transmembrane protein located at the cell surface [5], our genetic epistasis analysis (Figure 5E) suggests that SMA-10 is a negative regulator of the receptor DAF-2, which in turn is a repressor of the antimicrobial response directed by IIS.

In our experiments SMA-10 appears as both, an activator and an inhibitor of receptor kinases in *C. elegans*: activator of DAF-4/SMA-6 (Sma/Mab) [5,10] (Figure 1 and Figure 3) and repressor of DAF-2 (IIS; Figure 5E). Similarly, SMA-10’s mammalian homologs, LRIG1 and LRIG3, have been identified as negative and positive regulators, respectively, of receptor tyrosine kinases [9,35]. It would be useful to understand how the different domains of SMA-10 are interacting, directly or indirectly, positively or negatively, with its receptors, as has already been started to be grasped for LRIGs [35]. But the comparisons between nematodes and mammals should guide us not only into the specific biochemical interactions, but also looking for more general functional similarities, asking if LRIGs can modulate insulin or BMP receptors [5] or if SMA-10 can regulate other types of receptors that are known to be controlled by LRIGs in mammals [9]. Previously and closer to our results, further research should investigate the SMA-10/DAF-2 relationship; asking, for example, if it involves other kinds of genetic interaction within the IIS pathway, or if interactions are not only genetic but physical.

Finally, the proposal that SMA-10 may be working in both signalling pathways, TGF-β Sma/Mab and IIS, albeit leading to different phenotypes, opens the possibility of a molecular crosstalk between both pathways, as described for TGF-β Dauer and IIS [36,37].

## 4. Materials and Methods

### 4.1. Strains and Culture Conditions

Wild-type *Caenorhabditis elegans* N2 strain (Bristol) and the following mutant strains were obtained from *Caenorhabditis* Genetics Centre (CGC): AU37 *glp-4(bn2) I; sek-1(km4) X*, CB1370 *daf-2(e1370) III*, CB1482 *sma-6(e1482) II*, DA465 *eat-2(ad465) II*, EW15 *bar-1(ga80) X*, IG10 *tol-1(nr2033) I*, NU3 *dbl-1(nk3) V*, RB1739 *sma-10(ok2224) IV*, and RB911 *fshr-1(ok778) V*. Strains were cultured on agar plates seeded with *E. coli* OP50 and incubated at 20 °C according to standard procedures [38], except for CB1370 and AU37 that were grown at 15 °C. We generated combined mutants of RB1739 with the above strains (Table S4 for complete description of genotypes). The presence of each correspondent mutation was confirmed by polymerase chain reaction (PCR) and electrophoresis, and if necessary, by DNA sequencing (primers in Table S5).

### 4.2. Generation of Transgenic Strains for Tissue Specific SMA-10 Expression

A genomic fragment starting at the first codon of *sma-10* and including its 3′UTR was cloned into the nematode expression vector pPD95.77 (Andrew Fire collection, Addgene) by replacing the original GFP and 3′UTR of *unc-54*. To mimic the endogenous expression, a 1.6 kb genomic sequence upstream of the *sma-10* start codon was also cloned. For tissue-specific expression assays, the *sma-10* genomic coding region was cloned under the control of the *myo-2* promoter (pharyngeal muscle-specific expression [19]), *dpy-7* promoter (hypodermal expression [20]), or *trx-3* promoter (intestinal expression [21]) (see Table S5 for a list all the oligonucleotides used). Transgenic animals (Table S4) were created using standard microinjection techniques [39]. Each of the above constructs were microinjected at 20 ng/µL together with 20 ng/µL *P_sca-1_::gfp::3′UTR_unc-54_* (pGK10 plasmid [40]), 30 ng/µL of the hygromycin resistant vector pHygroSfi (a gift from Dr. J. Pérez-Martín) and 80 ng/µL 1 kb DNA ladder (Invitrogen).

### 4.3. Morphometrics

To calculate the length of adult worms, synchronized animals individually grown on 5 cm NGM Petri dishes were measured at 24 h intervals since around 90 h post-hatching until days after they reached plateau size. Final lengths were calculated from averages of worms measured two to three consecutive days after they had reached a plateau size. Images were captured using a video camera (JVC KY-F550, JVC Professional Europe Ltd.) attached to a dissecting microscope (×50, Leica MZ 7.5), and images were analysed with ImageJ software (1.46r; National Institutes of Health). A minimum of two independent experiments was carried out for each strain and treatment; with at least 20 animals per strain and experiment. Welch two-sample *t*-tests were used to assess statistical significance of differences in length between strains.

### 4.4. Hypodermal Ploidy

Upon completing growth, worms were fixed in Carnoy’s solution for a minimum of 24 h, stained with 100 ng/mL DAPI (4′,6-diamidino-2-phenylindole) for 30 min and observed under an AxioZeiss Imager.A1 (Zeiss, Oberkochen, Germany) epifluorescence microscope [41,42]. Nuclei images were collected using an Axiocam Mrm (Zeiss, Oberkochen, Germany) video camera with the Axiovision Release 4.8 software (Zeiss, Oberkochen, Germany) and analysed with ImageJ software. Diploid genome sizes were estimated from ventral cord cells, using DAPI-based densitometry [14,43]. A minimum of two independent experiments was carried out for each strain and treatment; with more than 10 animals per experiment and strain. Welch two-sample *t*-tests were used to assess statistical significance of differences in ploidy.

### 4.5. Lifespan Analysis

Lifespan analysis was performed at 20 °C in NGM plates with OP50 and 50 mM FUdR (2′-fluoro-5′-deoxy-uridine) to avoid worm progeny from developing and therefore to eliminate the matricide as previously described [16]. Synchronised L4 animals were transferred to plates (5 worms per plate) and survival monitored every 24 h. The first day of adulthood was defined as time zero. Animals that ruptured or crawled off the plates were included in the lifespan analysis as censored worms. Kaplan–Meier survival analysis was performed using GraphPad Prism version 5.01 software (La Jolla, CA, USA). In all cases, the Mantel-Cox (log-rank) test was used to assess statistical significance of differences in survival. Only *p* values smaller than 0.01 were considered significant. At least two independent experiments with a minimum of 50 animals per experiment were used.

### 4.6. Reproductive Span

Individual synchronized L4 hermaphrodites were moved to fresh NGM plates daily until reproduction ceased for at least two days. The last day of viable progeny production was noted as the day of reproduction cessation for each individual. When matricide occurred, the corresponding animal was censored from the experiment on that day. All experiments were performed at 20 °C. Two independent experiments were performed with at least 30 individuals per strain and experiment. The log-rank (Mantel–Cox) method was used to test the null hypothesis.

### 4.7. Progeny Production

Individual synchronized L4 hermaphrodites were moved to fresh plates and the number of progeny produced by each individual was counted daily until reproduction ceased for at least two days. When matricide occurred, the animal was censored from the experiment on that day. All experiments were performed twice at 20 °C with at least 30 individuals per strain and experiment. Welch two-sample *t*-tests were used for assessing differences.

### 4.8. Matricide

Assays were performed as in the analyses of reproductive span, and the cumulative percentage of hermaphrodites that underwent matricide was calculated daily. The matricide frequency was determined as the frequency of reproductive worms that die of matricide. For each strain, two replicas of 60 worms each were used.

### 4.9. Pathogenic Assays

Immunity assays were performed at 25 °C in Slow Killing (SK) plates (NGM with 3.5 g/L instead of 2.5 g/L of bactopeptone, and 20 g/L instead of 17 g/L of agar) with *Pseudomonas aeruginosa* PA14 as previously described [44,45]. 10 µL of a PA14 overnight culture in King’s broth (20 g/L proteose peptone 3; DIFCO, BD Diagnostics), 1.5 g/L KH_2_PO_4_, 15 mL pure glycerol, 6 mL/L 1M MgSO_4_ and rifampicin to a final concentration of 100 µg/mL) were spread onto SK plates and incubated overnight at 37 °C [46]. Ten L4 worms were transferred to each SK plate with PA14 bacteria and scored every 6–8 h for survival. Worms were transferred every two days to fresh plates. Animals that ruptured or crawled off the plates were censored. At least, two independent experiments with a minimum of 30 animals per experiment were analysed. Statistical analyses were performed as described for survival experiments above.

## Figures and Tables

**Table 1:** Effect of mutations on hypodermal ploidy.

Genotype	Hypodermal Ploidy	N	*p* Value
N2	10.86 ± 0.8	12	
*sma-10(ok2224)*	8.77 ± 0.7	14	0.0008 ^a^
*dbl-1(nk3)*	7.04 ± 0.3	17	<0.0001 ^a^, 0.0005 ^b^
*sma-10(ok2224);dbl-1(nk3)*	7.77 ± 0.6	12	<0.0001 ^a^, 0.067 ^b^, 0.065 ^c^

Hypodermal ploidy, mean with 95% confidence interval; N, sample size; *p* value, or probability of observing these differences between genotypes only due to chance, using the Welch two-sample *t*-test method. ^a^ Compared to N2. ^b^ Compared to *sma-10(ok2224)*. ^c^ Compared to *dbl-1(nk3)*.

## Data Availability

The data presented in this study are available in this article and the supplementary material.

## Supplementary Materials

Supplementary materials can be found at https://www.mdpi.com/1422-0067/22/2/638/s1.

## Abbreviations

TGF-β	Transforming growth factor beta
IIS	Insulin/insulin-like growth factor signalling
LRIG	Leucine-rich repeats and immunoglobulin-like domains
NGM	Nematode growth medium
