# Supplementary material for: SMA-10 Is a Non-Canonical Member of the TGF-β Sma/Mab Pathway and Immunity Regulator via the DAF-2 Insulin Receptor in Caenorhabditis elegans
Source: Int J Mol Sci. 2021 Jan 11;22(2):638. doi: 10.3390/ijms22020638 (PMC7827673; doi:10.3390/ijms22020638)
Supplement: Supplementary file 1 [file ijms-22-00638-s001.pdf]

International Journal of
Molecular Sciences

MDPI

Supplementary Information for:

SMA-10 is a non-canonical member of the TGF-β Sma/Mab pathway and immunity regulator via the DAF-2 insulin receptor in *Caenorhabditis elegans*

María Pilar de Lucas [1], Marta Jiménez [1], Paloma Sánchez-Pavón [1], Alberto G. Sáez [1] and Encarnación Lozano [1,*]

[1] Unidad Funcional de Investigación de Enfermedades Crónicas, Instituto de Salud Carlos III, 28220 Majadahonda, Madrid, Spain.; mpdelucas@isciii.es (M.P.L.); marta.jimenezsanchez90@gmail.com (M.J.); paloma.sanchezpavon@gmail.com (P.S.); agsaez@gmail.com (A.G.S.)

* Correspondence: encarnilozano@gmail.com

Keywords: *sma-10*; TGF-β Sma/Mab; TGF-β signalling; IIS pathway; *daf-2*; immune response.

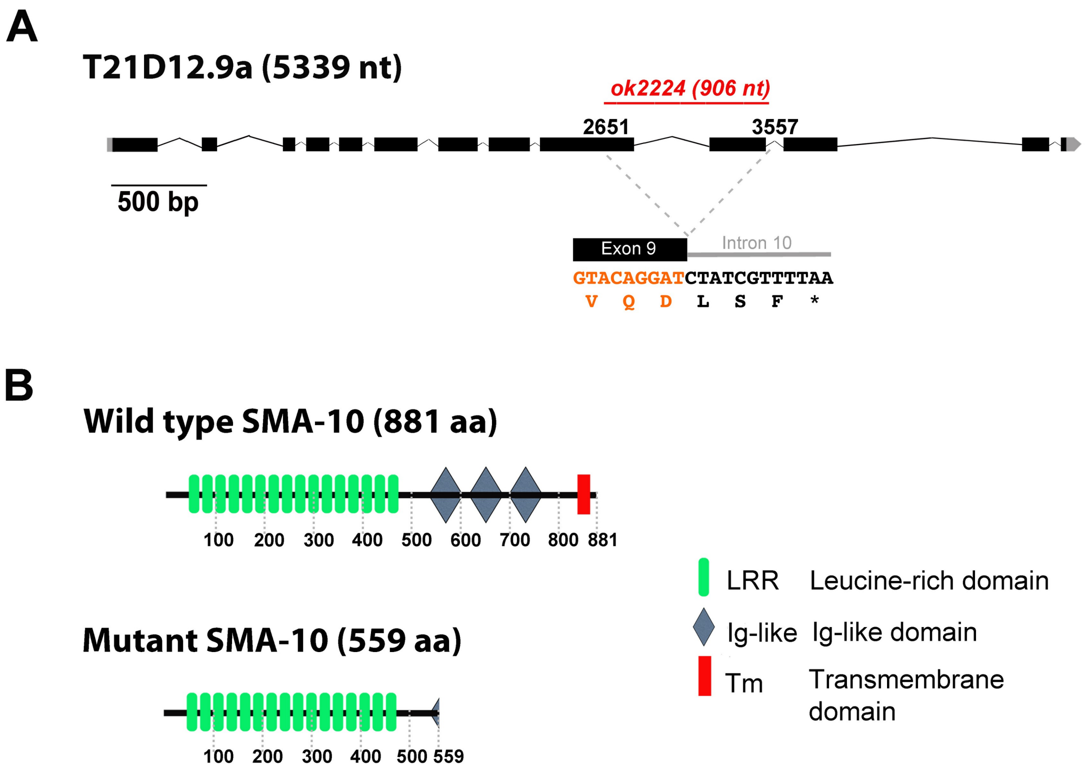

Figure S1. Schematic representation of the T21D12.9a transcript and its wild-type and mutant SMA-10 proteins. (**A**) T21D12.9a transcript is composed of 13 exons and spans about 5.4 kb, including the 5′ and 3′UTRs. The red line marks the location of the *ok2224* deletion, that removes 906 nucleotides (from positions 2651 to 3557) corresponding to part of the exon 9, the whole intron 9 and exon 10, and the start of the intron 10. This deletion creates a new stop codon in the spliced transcript and, therefore, a truncated SMA-10 when it is transcribed. (**B**) Schematic illustration of wild-type SMA-10 with sixteen annotated predicted leucine rich regions (LRR in green), three Ig-like domains (in grey) and the transmembrane region (red). The mutant SMA-10 is only composed by 559 amino acids and lacks almost the entire three Ig-like domains, the transmembrane region and the short cytoplasmic portion of the protein. Numbers specify positions of amino acids.

Table S1. Adult body length of *sma-10* mutants and other body-size related genotypes of *C. elegans*.

Genotype	Length (mm)	N	*P* value
N2	1.245	84	
sma-10(ok2224)	0.897	92	< 0.001[a]
dbl-1(nk3)	0.808	55	< 0.001[a], < 0.001[b]
sma-10(ok2224);dbl-1(nk3)	0.781	54	< 0.001[a], < 0.001[b], 0.004[c]
eat-2(ad465)	0.895	20	< 0.001[a], 0.716[b], < 0.001[c]
sma-10(ok2224);eat-2(ad465)	0.621	20	< 0.001[a], < 0.001[b], < 0.001[d]

Length, measured as final mean length of at least two independent experiments; N, sample size; *P* value, or probability that the observed differences between genotypes were due to chance, using the Welch Two Sample t-test. [a] Compared to N2 wild-type. [b] Compared to *sma-10(ok2224).* [c] Compared to *dbl-1(nk3).* [d] Compared to *eat-2(ad465).*

Table S2. Effect of different genetic backgrounds and tissue-dependent *sma-10* expression on survival on PA14 pathogenic bacteria.

Genotype	Mean Survival (hours)	N	*P* value
N2	90	40	
sma-10(ok2224)	68	40	0.0009[a]
dbl-1(nk3)	65	40	< 0.0001[a], 0.4625[b]
sma-10(ok2224);dbl-1(nk3)	40	40	< 0.0001[a], < 0.0001[b], < 0.0001[c]
N2	116	24	
sma-10(ok2224)	81	30	< 0.0001[a]
$P_{sma\text{-}10}$::*sma-10*	120	30	0.5195[a], < 0.0001[b]
N2	116	24	
sma-10(ok2224)	70	60	< 0.0001[a]
$P_{sma\text{-}10}$::*sma-10*	116	47	0.3363[a]
$P_{trx\text{-}3}$::*sma-10*	116	60	0.8838[a], 0.3414[b]
$P_{dpy\text{-}7}$::*sma-10*	122	56	0.0813[a], 0.3459[b]
$P_{myo\text{-}2}$::*sma-10*	77	54	< 0.0001[a], < 0.0001[b]

Mean Survival (hours), average length of time that worms survive on a *Pseudomonas aeruginosa* (strain PA14) lawn; N, sample size; *P* value, probability that the observed differences between genotypes were due to chance, using the Log-rank (Mantel-Cox) test. [a] compared to the concurrent N2 set. [b] compared to *sma-10(ok2224).* [c] compared to *dbl-1(nk3).*

Table S3. Effect of different genetic backgrounds on survival on PA14 pathogenic bacteria.

Genotype	Mean Survival (hours)	N	*P* value
N2	115	40	
sma-10(ok2224)	91	40	< 0.001[a]
sek-1(km4)	48.5	40	< 0.001[a]
sma-10(ok2224);sek-1(km4)	42	40	< 0.001[b]
N2	93	40	
sma-10(ok2224)	69	40	< 0.001[a]
fshr-1(ok778)	42	40	< 0.001[a]
sma-10(ok2224);fshr-1(ok778)	17	40	< 0.001[c]
N2	117	40	
sma-10(ok2224)	73	40	< 0.001[a]
bar-1(ga80)	70	40	< 0.001[a]
sma-10(ok2224);bar-1(ga80)	21	41	< 0.001[d]
N2	115	40	
sma-10(ok2224)	91	40	< 0.001[a]
tol-1(nr2033)	115	40	0.52[a]
sma-10(ok2224);tol-1(nr2033)	91	40	0.89[e]
N2	93	40	
sma-10(ok2224)	68	40	< 0.001[a]
daf-2(e1370)	135	38	< 0.001[a]
daf-2(e1370);sma-10(ok2224)	140	40	0.45[f]

Mean Survival (hours), average length of time that worms survive on a *Pseudomonas aeruginosa* (strain PA14) lawn; N, sample size; *P* value, probability of having those differences between genotypes only because of chance, using the Log-rank (Mantel-Cox) test. [a] Compared to concurrent N2. [b] Compared to *sek-1(km4)*. [c] Compared to *fshr-1(ok778)*. [d] Compared to *bar-1(ga80)*. [e] Compared to *sma-10(ok2224)*. [f] Compared to *daf-2(e1370)*.

Table S4. *C. elegans* strains generated in this study.

Strain Name	Genotype
ENL95	*sma-10(ok2224) IV* backcrossed 6x
ENL59	*sma-10(ok2224) IV; dbl-1(nk3) V*
ENL64	*sma-10(ok2224) IV; fshr-1(ok778) V*
ENL65	*sma-10(ok2224) IV; sek-1(km4) X*
ENL66	*eat-2(ad465) II; sma-10(ok2224) IV*
ENL69	*tol-1(nr2033) I; sma-10(ok2224) IV*
ENL70	*daf-2(e1370) III; sma-10(ok2224) IV*
ENL96.1	*sma-10(ok2224) IV; madEx30 [pGK10 (P_{sca-1}::gfp::3′UTR$_{unc-54}$) + pHygroSfi)]*
ENL96.2	*sma-10(ok2224) IV; madEx30 [pGK10 (P_{sca-1}::gfp::3′UTR$_{unc-54}$) + pHygroSfi)]*
ENL98.1	*sma-10(ok2224) IV; madEx31 [P_{myo-2}::genomic sma-10::3′UTR$_{sma-10}$ + pGK10 (P_{sca-1}::gfp::3′UTR$_{unc-54}$) + pHygroSfi)]*
ENL98.2	*sma-10(ok2224) IV; madEx31 [P_{myo-2}::genomic sma-10::3′UTR$_{sma-10}$ + pGK10 (P_{sca-1}::gfp::3′UTR$_{unc-54}$) + pHygroSfi)]*
ENL98.3	*sma-10(ok2224) IV; madEx31 [P_{myo-2}::genomic sma-10::3′UTR$_{sma-10}$ + pGK10 (P_{sca-1}::gfp::3′UTR$_{unc-54}$) + pHygroSfi)]*
ENL99	*sma-10(ok2224) IV; madEx32 [P_{dpy-7}::genomic sma-10::3′UTR$_{sma-10}$ + pGK10 (P_{sca-1}::gfp::3′UTR$_{unc-54}$) + pHygroSfi)]*
ENL100.1	*sma-10(ok2224) IV; madEx33 [P_{trx-3}::genomic sma-10::3′UTR$_{sma-10}$ + pGK10 (P_{sca-1}::gfp::3′UTR$_{unc-54}$) + pHygroSfi)]*
ENL100.2	*sma-10(ok2224) IV; madEx33 [P_{trx-3}::genomic sma-10::3′UTR$_{sma-10}$ + pGK10 (P_{sca-1}::gfp::3′UTR$_{unc-54}$) + pHygroSfi)]*
ENL101.1	*sma-10(ok2224) IV; madEx34 [P_{sma-10}::genomic sma-10::3′UTR$_{sma-10}$ + pGK10 (P_{sca-1}::gfp::3′UTR$_{unc-54}$) + pHygroSfi)]*
ENL101.2	*sma-10(ok2224) IV; madEx34 [P_{sma-10}::genomic sma-10::3′UTR$_{sma-10}$ + pGK10 (P_{sca-1}::gfp::3′UTR$_{unc-54}$) + pHygroSfi)]*
ENL101.3	*sma-10(ok2224) IV; madEx34 [P_{sma-10}::genomic sma-10::3′UTR*$_{\text{sma-10}}$ + *pGK10 (P_{sca-1}::gfp::3′UTR$_{unc-54}$) + pHygroSfi)]*
ENL103	*sma-10 (ok2224) IV; bar-1(ga80) X*

Table S5. Primers for selecting double mutants and for generating strains with transgenic *sma-10* expression.

Genotype	Primer sequence	Orientation
sek-1(km4)	5′-CATCCATACACTAGAATAAGTG	forward
	5′-GTGAAGTGTGCTTAAATTGC	forward
	5′-CAGATGCTAATAAACTACTTGAAC	reverse
bar-1(ga80)	5′-TCTTACATTATCCTGATCTTTC	forward
	5′-ACAGGGAGCAACACACCTCGAG	reverse
sma-10(ok2224)	5′-ATCATGGCTCGCATCGTGGA	forward
	5′-GAAGATCAAGGAGCATACAC	forward
	5′-CTTCTTCCACTACCCCGAAA	reverse
daf-2(e1370)	5′-CGACAACCTGATCATTCATATC	forward
	5′-CTGTCAAGATTGGAGATTTCGG	reverse
tol-1(nr2033)	5′-CGATTGCTGTTCTCCTCTGTG	forward
	5′-CCTCGTGAATAATGGAAACTCG	reverse
fshr-1(ok778)	5′-GGGATTGATTGTGTCTCAATTGC	forward
	5′-AGGTGGTTGAGAATGATAGAAG	reverse
	5′-ACCATGACGCTCCCACCTC	reverse
dbl-1(nk3)	5′-CGACTCTGTGCGGACAACTA	forward
	5′-AAGCATCGTAGCCCTCTGAA	reverse
	5′-CATGGACAAACATCGGGGA	forward
	5´-CGTGTACACAAATCTGTTCG	reverse
eat-2(ad465)	5′-TGATCACCCTAGTTGTCTGG	forward
	5′-AGTGTAGAGGTACTGTATGG	reverse
sma-10 (genomic)	5´-ATGATTGTATATTCGCTATTAC	forward
	5´-TTTAACAATTATAAGTATTTAC	reverse
sma-10 (promoter)	5′-GATGGAGGTTTTACGGTTTTCGG	forward
	5′-GTCCACATTTTTGTCACAGAAAAGG	reverse
dpy-7 (promoter)	5′-GAAATGGTGAGTGTTCGAGCTC	forward
	5′-TTATCTGGAACAAAATGTAAGAA	reverse
myo-2 (promoter)	5′-TCGAGGCATTTGAATTGGGG	forward
	5′-CGAGGGTTAAAATGAAAAGTGGTGG	reverse
